# Zosurabalpin: a novel tethered macrocyclic peptide antibiotic that kills carbapenem‐resistant *Acinetobacter baumannii*

**DOI:** 10.1002/mco2.696

**Published:** 2024-08-11

**Authors:** Qi Weng, Feng Zhang, Quan Zheng

**Affiliations:** ^1^ Department of Pharmacy The Quzhou Affiliated Hospital of Wenzhou Medical University, Quzhou People's Hospital Quzhou China; ^2^ Core Facility The Quzhou Affiliated Hospital of Wenzhou Medical University, Quzhou People's Hospital Quzhou China

1

In two recent studies published back‐to‐back in *Nature*, Bradley's and Kahne's teams reported a novel tethered macrocyclic peptide (MCP) antibiotic called zosurabalpin, which targets the LptB2FGC complex in the inner membrane to block lipopolysaccharide (LPS) transport, leading to the accumulation of this endotoxin in the cell, ultimately resulting in the death of the bacteria (Figure 1).^1^, ^2^ Zosurabalpin not only showed excellent antibacterial activity against carbapenem‐resistant *Acinetobacter baumannii* (CRAB) in vivo and in vitro, but is also expected to break through the resistance mechanisms of existing antibiotics, offering new prospects for clinical treatment.

**FIGURE 1:**
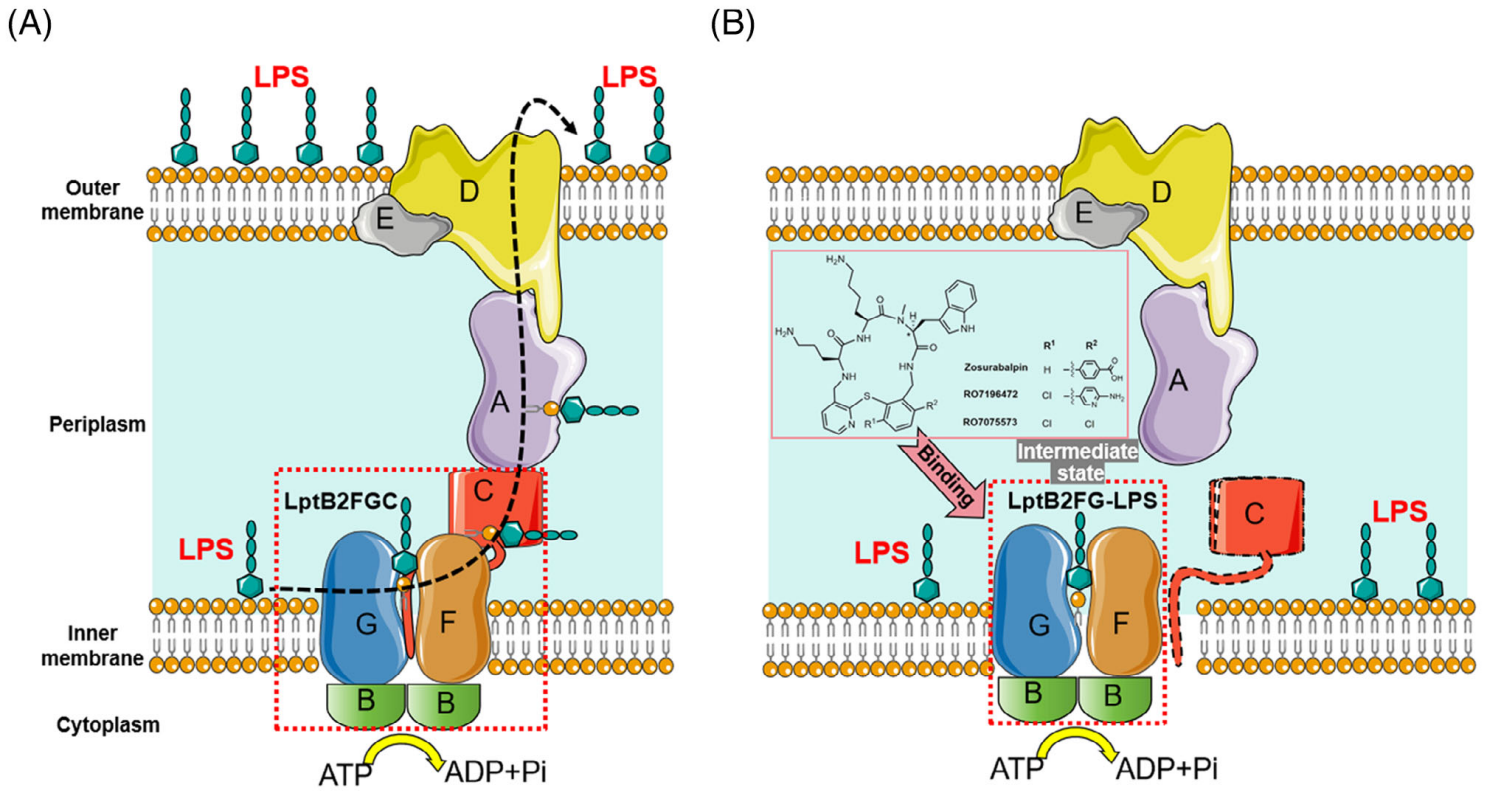
A) Mechanisms of lipopolysaccharide (LPS) transport. The Lpt multiprotein complex consists of three parts: the inner membrane transporter protein complex LptB2FGC, the membrane interstitial protein LptA, and the outer membrane protein complex LptDE. LPS is extracted from the inner membrane by LptB2FG and then transported to the outer side of the outer membrane via a transport bridge consisting of LptC, LptA, and LptDE. (B) Mechanisms of antimicrobial action of the novel antibiotic. When the LptC transmembrane (TM) helix dissociates from the LptB2FG complex, it allows zosurabalpin to bind to the intermediate state of LptB2FG–LPS to form a ternary complex, which blocks the transport of LPS, leading to the intracellular accumulation of this endotoxin and ultimately to bacterial death.

*A. baumannii*, a Gram‐negative bacterium with an outer membrane containing LPS, is resistant to penetration by a wide range of antibiotics, making it difficult to kill. It is one of the common causative agents of hospital‐acquired infections and results in hospital‐acquired pneumonia, bloodstream infections, urinary tract infections, and soft tissue skin infections. In recent years, with the extensive use of antibiotics, the resistance of *A. baumannii* has been increasing, bringing great challenges to clinical treatment. One of the infections caused by CRAB is particularly difficult to treat, which is listed as a priority on the World Health Organization's list of key pathogens and also as an urgent threat by the Centers for Disease Control and Prevention in the United States.^3^ Therefore, the development of novel antibiotics against Gram‐negative bacteria has become an urgent research topic for researchers.

In order to find novel antibiotics against CRAB, Bradley's team screened and optimized 44,985 MCPs produced by Tranzyme Pharma for antimicrobial activity (both Gram‐negative and Gram‐positive), and identified the leading compound, RO7075573, which showed significant inhibition of *A. baumannii* with a minimum inhibitory concentration (MIC) of 0.12 mg L^−1^.^1^ It also demonstrated high selectivity and was ineffective against wild‐type, exocytosis‐impaired, and pore‐protein‐deficient *Escherichia coli*, *Klebsiella pneumoniae*, and *Pseudomonas aeruginosa*. In addition, RO7075573 showed similar antimicrobial activity against antibiotic‐sensitive strains and multidrug‐resistant *A. baumannii* strains, suggesting that its mechanism of action is different from that of the current clinically applied antibiotics and that it may overcome existing resistance mechanisms. However, severe tolerance issues were observed in rat models of intravenous administration. Therefore, the researchers optimized the structure of the MCPs, and successfully acquired an amphoteric benzoic acid derivative, zosurabalpin. It showed an MIC of 0.25 mg L^−1^ against *A. baumannii*, and demonstrated superior tolerability.

To identify potential molecular targets of zosurabalpin, *A. baumannii* was induced to spontaneously develop resistance by incrementally increasing the concentration of zosurabalpin in the medium, which was then analyzed by gene sequencing to reveal mutation sites. The researchers found 28 differential mutations in the gene encoding LptF and two unique mutations in LptG. As LptF and LptG are components of the LptB2FGC complex, which is a key part of the LPS transport system, this suggests that zosurabalpin may target the LptB2FGC complex to block LPS transport. In addition, zosurabalpin not only showed good pharmacokinetics with high clearance (51 mL min^−1^ kg^−1^), low volume of distribution (0.7 L kg^−1^), short terminal half‐life (0.3 h), and moderate protein binding (fraction unbound, 37%), but also demonstrated good in vivo efficacy in mouse models of infection, including sepsis and femur and lung infections caused by CRAB strains. Furthermore, phase I clinical studies have demonstrated that a single intravenous dose of 10–2000 mg of zosurabalpin is generally safe and well tolerated.^4^

Meanwhile, another study elucidated for the mechanism of the antibacterial activity of tethered MCP antibiotics (including zosurabalpin, RO7196472 and RO7075573).^2^ First, Kahne's team observed by cryo‐electron microscopy (cryo‐EM) that RO7196472 binds to LptB2FG as well as LPS to form a ternary complex, in which RO7196472 binds to pockets formed by the arrangement of the side chains of several amino acids in the transmembrane (TM) helices of LptF (Glu58, Glu249, Trp271, Val314, Ile317, Arg320, and Thr321) and LptG (Leu36). More importantly, Kahne's team also observed that RO7196472 captures an intermediate state of LptB2FG–LPS. However, the overall conformation of LptB2FG–LPS remained essentially stable with or without RO7196472, suggesting that the cyclic peptide binds to the pre‐existing structure of the LptB2FG–LPS complex, thus confirming that this state is a druggable conformation for antibiotic development.

LptC is a member of the LptB2FGC complex, and plays an important role in the transfer of LPS from LptF to LptA (Figure 1A).^5^ By analyzing the cryo‐EM structures of zosurabalpin, RO7196472, and RO7075573 bound to the LptB2FG–LPS complex while comparing the efficacy of these three MCP antibiotics, Kahne's team found that the MCP‐targeted LptC TM helix had dissociated from the complex (Figure 1B). In addition, the results of ATPase activity and LPS release assays further validated that the compounds had the strongest affinity for LptB2FG–LPS in the absence of LptC. However, the researchers found that *A. baylyi* strains lacking LPS in the outer membrane could grow in vitro in the presence of high concentrations of RO7196472, suggesting that MCPs do not act by depleting LPS from the outer membrane, but rather by its toxic accumulation within the cell. As LPS is a key component of the outer membrane of Gram‐negative bacteria, its abnormal intracellular accumulation may interfere with normal intracellular physiological activities, including enzyme activity, the conduct of metabolic pathways, and cell signaling. When LPS accumulates to a certain level, it may trigger intracellular death procedures, such as activation of autolysin or induction of apoptosis, leading to cell death. Therefore, it is necessary to further explore the specific mechanism by which zosurabalpin contributes to the intracellular accumulation of LPS, and thus induces cell death. On the one hand, we can analyze the changes in gene expression and protein expression in zosurabalpin‐treated bacteria to identify the molecular pathways involved in response to LPS accumulation. On the other hand, we can investigate the effects of LPS accumulation on bacterial metabolic pathways, especially those related to energy production, amino acid synthesis, and lipid metabolism.

In summary, zosurabalpin, with its distinctive mechanism of action, selective antibacterial activity, highly potent in vitro and in vivo efficacy, and favorable tolerability, exhibits notable competitive advantages in the domain of CRAB therapy. First, zosurabalpin is highly selective for CRAB and has limited activity against other Gram‐negative and Gram‐positive bacteria, helping to minimize the impact on normal flora and thus potentially reducing associated side effects. Second, due to the novel mechanism of action of zosurabalpin, CRAB currently lacks an effective defense mechanism in the face of zosurabalpin, which will provide a new option for the clinical treatment of drug‐resistant strains. However, researchers have found that selective mutations in the LPS transport mechanism can reduce the potency of the drug, implying that *Acinetobacter* strains will inevitably develop resistance to this class of antibiotics as well. Consequently, it is imperative to investigate the potential of combining zosurabalpin with other antibiotics to enhance therapeutic efficacy and impede the emergence of resistance. Meanwhile, it is essential to investigate the mechanisms by which Gram‐negative bacteria develop resistance to zosurabalpin, as well as the means of preventing and reversing such resistance. In addition, the LPS transporter LptB2FGC complex, as a new potential antibiotic target, provides ideas for the future development and design of synthetic novel antibiotics. We can also further chemically modify MCPs to improve their stability, bioavailability, and reduce potential side effects.

## AUTHOR CONTRIBUTIONS

**Quan Zheng** conceived the manuscript. **Qi Weng** and **Quan Zheng** wrote the manuscript. **Qi Weng** prepared the figure. **Feng Zhang** proofread and edited the manuscript. All authors have read and approved the article.

## CONFLICT OF INTEREST STATEMENT

The authors declare they have no conflicts of interest.

## ETHICS STATEMENT

Not applicable.

## Data Availability

Not applicable.
